# Research hotspots and frontiers of endoplasmic reticulum in glomerular podocytes: a bibliometric and visual analysis from 2005 to 2023

**DOI:** 10.3389/fphar.2024.1488340

**Published:** 2025-01-07

**Authors:** Wang Junli, Hao Zhihong, Wang Lina, Ou Qiaoqun, Qu Jing, Hu Jiaqi, Shengyou Yu

**Affiliations:** ^1^ Department of Pediatrics, The Second Affiliated Hospital, South China University of Technology, Guangzhou, China; ^2^ Department of Pediatrics, Guangzhou First People’s Hospital, School of Medicine, South China University of Technology, Guangzhou, Guangdong, China; ^3^ Department of Pediatrics, Guangzhou First People’s Hospital, Guangdong Medical University, Zhanjiang, Guangdong, China

**Keywords:** glomerular podocyte, endoplasmic reticulum, research hotspots, endoplasmic reticulum stress, bibliometric analysis

## Abstract

**Background:**

The glomerular podocyte endoplasmic reticulum is a critical component in renal function, yet its research landscape is not fully understood. This study aims to map the existing research on podocyte endoplasmic reticulum by analyzing publications in the Web of Science Core Collection (WOSCC) from the past 19 years.

**Methods:**

We conducted a bibliometric analysis using Citespace, VOSviewer, the Metrology Literature Online platform, and the Bibliometrix software package to visualize and interpret the data from WOSCC. The analysis focused on publication volume, authorship, institutional contributions, and research trends.

**Results:**

The analysis revealed a significant growth in publications, indicating a surge in interest in podocyte endoplasmic reticulum research. Cybulsky, Andrey V, and Papillon, Joan emerge as the most prolific authors, and the Journal of the American Society of Nephrology is the leading journal in this field. China is the top contributor in terms of publications, with McGill University being the most productive institution. The research primarily focuses on endoplasmic reticulum stress, diabetic nephropathy, and apoptosis, with emerging trends in “foot cell apoptosis,” “cell signaling pathways,” and “autophagy.”

**Conclusion:**

The findings highlight the expanding scope of podocyte endoplasmic reticulum research, with a particular emphasis on the mechanisms of endoplasmic reticulum stress and podocyte apoptosis. Future research directions may include the identification of specific therapeutic targets, detailed exploration of podocyte signaling pathways, and the role of autophagy. This study provides a comprehensive overview of the major research areas, frontiers, and trends in podocyte endoplasmic reticulum research, which are pivotal for guiding future investigations.

## 1 Introduction

**FIGURE 1:**
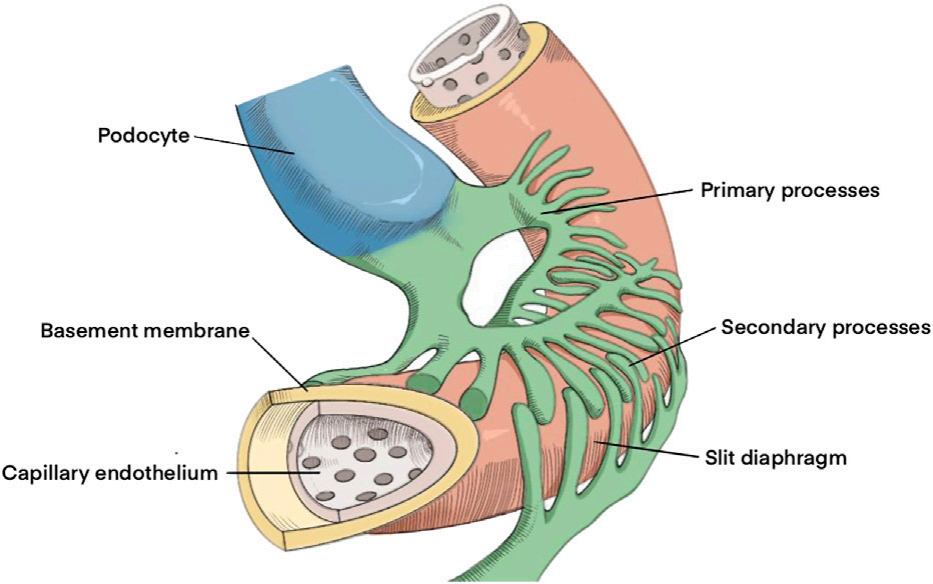
Structure of GBM and podocyte.

The glomerular filtration barrier is composed of glomerular basement membrane (GBM), endothelial and epithelial cells. The epithelial cells are known as podocytes, which are highly specialized and consist of cell bodies, primary processes and branching podocytes (Lin and Susztak, 2016). Podocytes extend many foot processes, interdigitated with each other, the top of the foot process and the loose layer outside the basement membrane contact (Figure 1). Between the foot processes, there is a filtering slit, and this slit is covered by a layer of slit diaphragm. The endoplasmic reticulum (ER), one of the largest organelles in eukaryotic cells, consists of a network of tubules and flattened sacs. These structures are connected by enclosed spaces known as the endoplasmic reticulum lumen, which is isolated from the surrounding cytoplasm by a single lipid bilayer, the endoplasmic reticulum membrane (Oakes and Papa, 2015). The endoplasmic reticulum plays an essential role in protein synthesis, folding, and structural maturation. Protein processing procedures within the endoplasmic reticulum include signal sequence cleavage, N-linked glycosylation, disulfide bond formation, isomerization or reduction [catalyzed by protein disulfide isomerase (PDI), oxidoreductase], proline isomerization, or lipid coupling, all of which ultimately result in a properly folded conformation (Almanza et al., 2018). The unique function of the endoplasmic reticulum is critical for the cell. Various injuries, such as viral infections, inflammation, toxic substances, and gene mutations, can impair endoplasmic reticulum function. Due to the large capacity of the endoplasmic reticulum of the podocyte, the level of anabolic or catabolic activity is high (Roumeliotis et al., 2023); thus the podocytes are susceptible to the endoplasmic reticulum, which affects the glomerular filtration barrier. Podocyte injury leads to proteinuria, a hallmark of most glomerular diseases (Pavenstädt et al., 2003). Renal diseases in which direct or indirect foot cell injury results in proteinuria or nephrotic syndrome are called podocytopathy (Kopp et al., 2020). So far, there has been no bibliometric study on the research progress of the endoplasmic reticulum of podocytes, and the statistical analysis using bibliometric methods in this paper can provide a more comprehensive understanding of the research hotspots and trends of the endoplasmic reticulum of podocytes, and provide some critical information for the direction of future research in this field.

## 2 Materials and methods

### 2.1 Retrieval strategy

**FIGURE 2:**
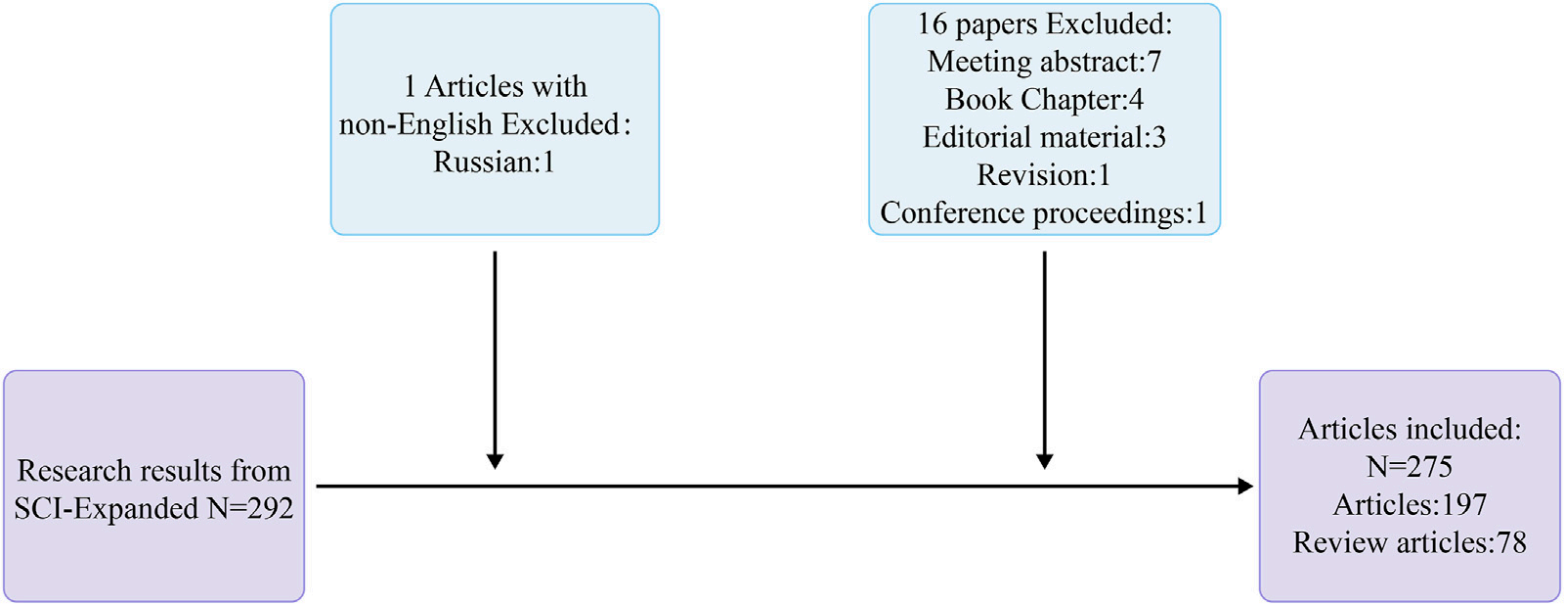
Flowchart of literature retrieval.

A search was performed within the Web of Science Core Collection database, resulting in literature that was published between 1 January 2005, and 22 October 2023. To ensure the consistency of the statistics, we searched on 22 October 2023, and downloaded the literature. We created two search formulas: #1 Subject term = (Podocyte OR Glomerular Visceral Epithelial Cells); #2 Subject term = (Endoplasmic Reticulum OR Ergastoplasm). The final search strategy consisted of combining criteria #1 and #2. The search terms used were derived from the Medical Subject Headings (MeSH) published by PubMed. The resulting literature was restricted to English-language papers and reviews (Figure 2).

### 2.2 Data analysis

Two team members independently downloaded and analyzed the literature, excluding any duplicate studies. Eligible literature, which met the study’s criteria, was then exported in plain text format, encompassing the full record and all cited references. For this study, we primarily utilized VOSViewer version 1.6.19, the Bibliometrics Online Analysis Platform, and the Bibliometrix software package to analyze and visualize the data. We used VOSviewer to do the following analyses: the publication of countries/institutions, co-authorship analysis of authors, journals and co-cited journals, co-citation analysis of references and co-occurrence analysis of keywords. We used the Citespace to analyze citation bursts of references and keywords.We used the Bibliometrics Online Analysis Platform to analyze the contribution of publications and national partnerships. We used the Bibliometrix software package to analyze the region of issuance and keyword dynamics.

#### 2.2.1 Trends in publications

**FIGURE 3:**
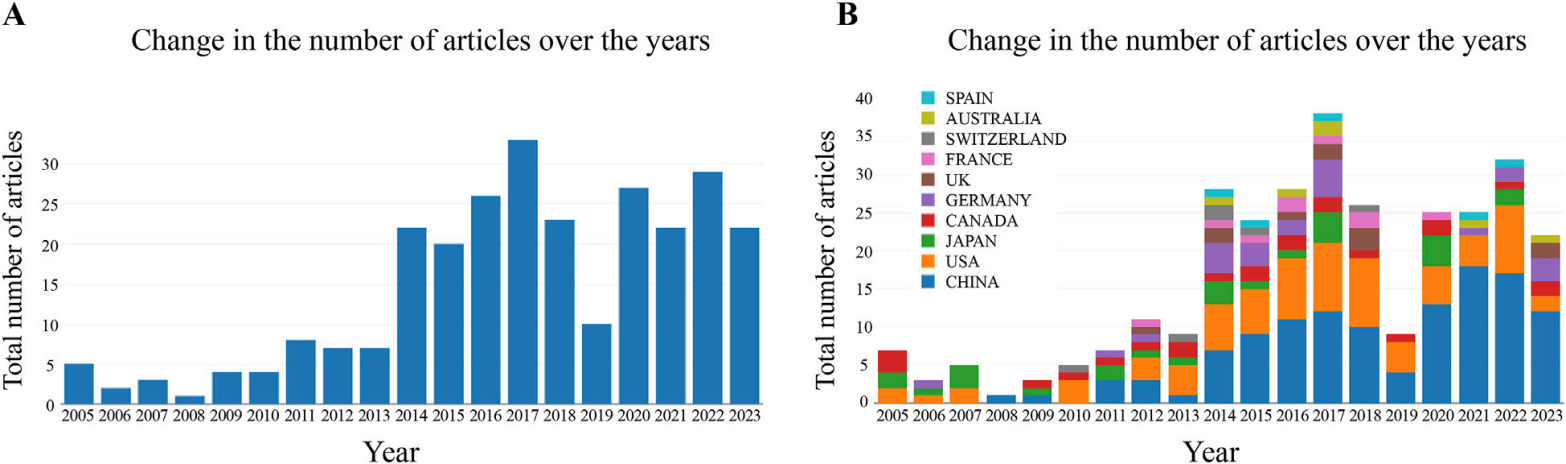
**(A)** Change in the number of articles over the years; **(B)** Change in the number of articles over the years (by country).

We searched a total of 275 documents, containing 197 papers and 78 reviews. As shown in Figure 3A, the number of global publications on the endoplasmic reticulum of glomerular podocytes has shown an upward trend in the past 19 years, with the number of publications increasing from 5 in 2005 to 22 in 2023. Between 2005 and 2013, research in this field was in its initial stage, and the number of publications grew slowly. From 2014, the number of papers published in the field increased dramatically, reaching 33 in 2017. In recent years, the worldwide number of publications has stabilized at 25 papers/year. The upward trend in the global number of publications indicates that the research on the endoplasmic reticulum of podocytes has become a focus of attention and has entered an accelerated period of development. Figure 3B reveals that China and the United States account for a significant portion of the global publication count, suggesting that these two countries have conducted a substantial amount of research in this field.

#### 2.2.2 Analysis of country and agency cooperation

**FIGURE 4:**
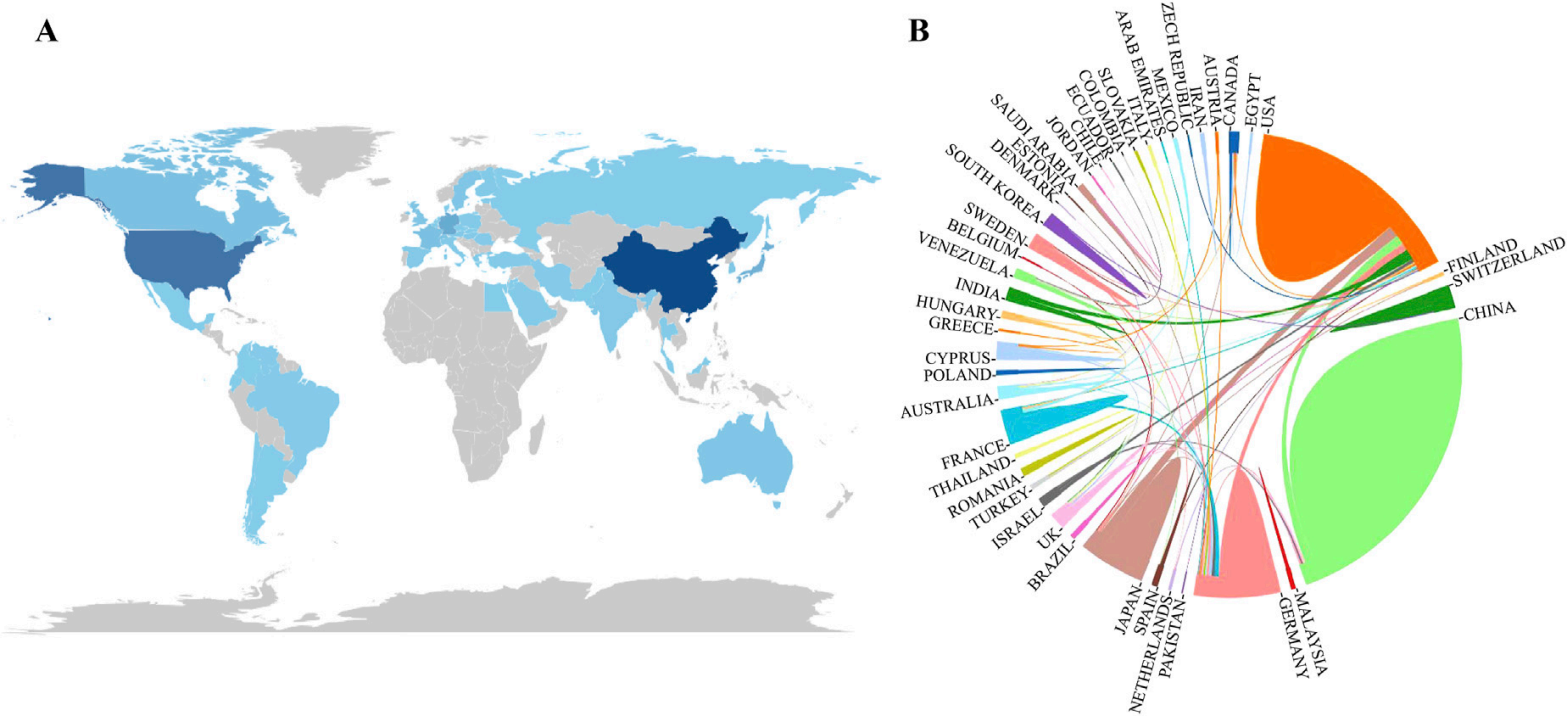
**(A)** Distribution of the number of articles issued by countries; **(B)** Analysis of the degree of cooperation and contribution of countries/regions. Note: **(A)** The darker the color of the country, the higher the number of publications; **(B)** The larger the area occupying the circle, the higher the number of articles issued; the thicker the line, the stronger the cooperation.

Forty-six countries have published papers in research on the endoplasmic reticulum of glomerular podocytes, and the countries with the highest number of publications are China and the United States. Table 1 shows the 10 countries and institutions with the highest number of publications in the past 19 years, and the top three countries with the highest number of publications are China (119), the United States (77), and Japan (26), this can also be seen in Figure 4A. In the analyzing of the cooperation relationship between countries, the closest cooperation was between China and the United States, and between the United States, and Japan (Figure 4B). Four hundred thirty-seven nstitutions published papers in the research on the endoplasmic reticulum of glomerular peduncle cells, and a total of 7 of the 10 institutions with the highest number of publications over the past 19 years were in China. The top three institutions with the highest number of publications were McGill University (21), Fudan University (10), and Huazhong University of Science and Technology (9).

**TABLE 1:** Top 10 most productive countries, institutions.

Rank	Nations	Publications	Rank	Organizations	Publications
1	China	119	1	Mcgill Univ (Canada)	21
2	United States	77	2	Fudan Univ (China)	10
3	Japan	26	3	Huazhong Univ Sci & Technol (China)	9
4	Canada	23	4	Shanghai Jiao Tong Univ (China)	9
5	Germany	23	5	Washington Univ (United States)	8
6	England	11	6	Cent South Univ (China)	7
7	France	9	7	Capital Med Univ (China)	6
8	Australia	6	8	China Acad Chinese Med Sci (China)	6
9	Switzerland	6	9	Kanazawa Med Univ (Japan)	6
10	India	5	10	Nanjing med univ (China)	6

#### 2.2.3 Authors’ analysis

**FIGURE 5:**
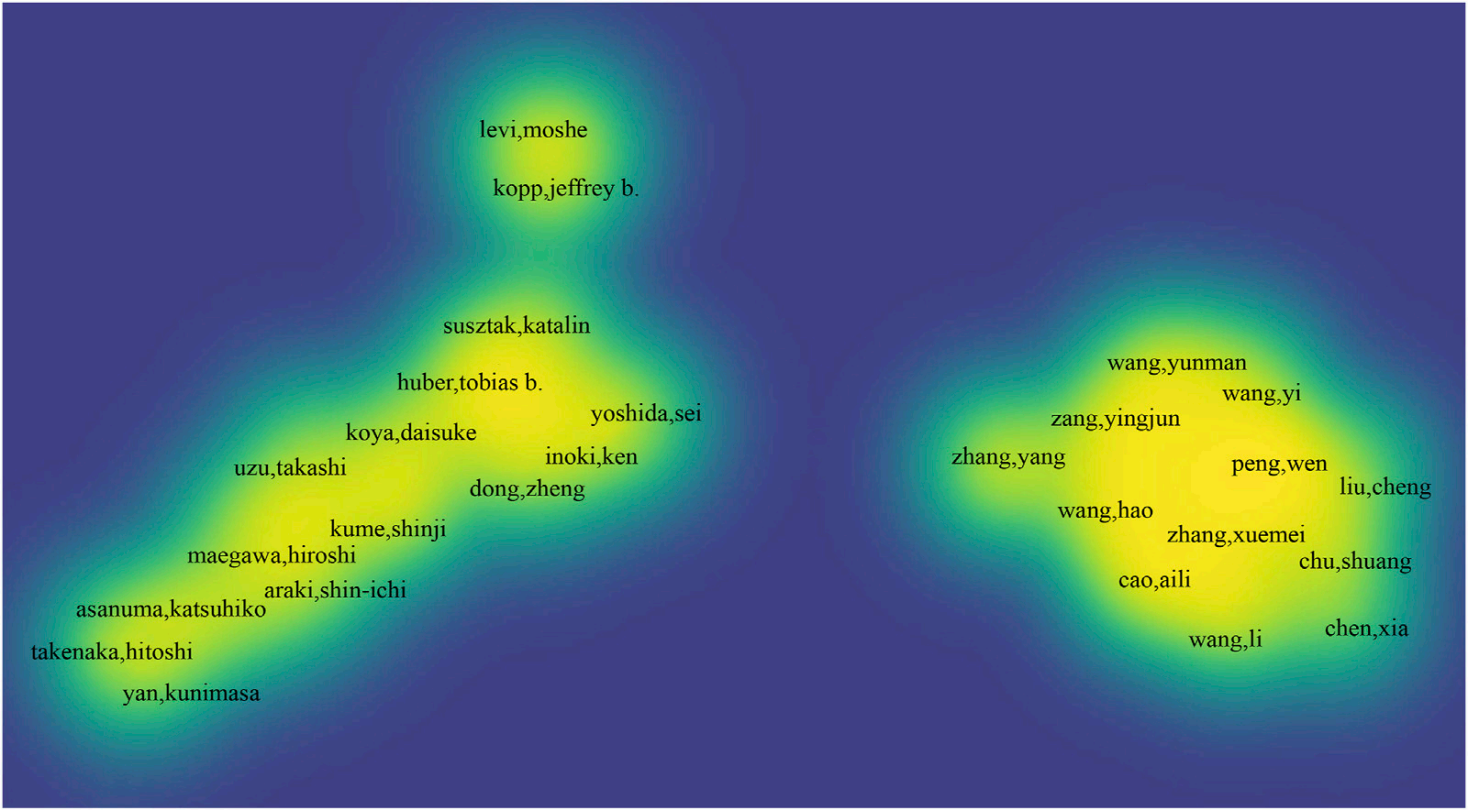
Author density map.

**FIGURE 6:**
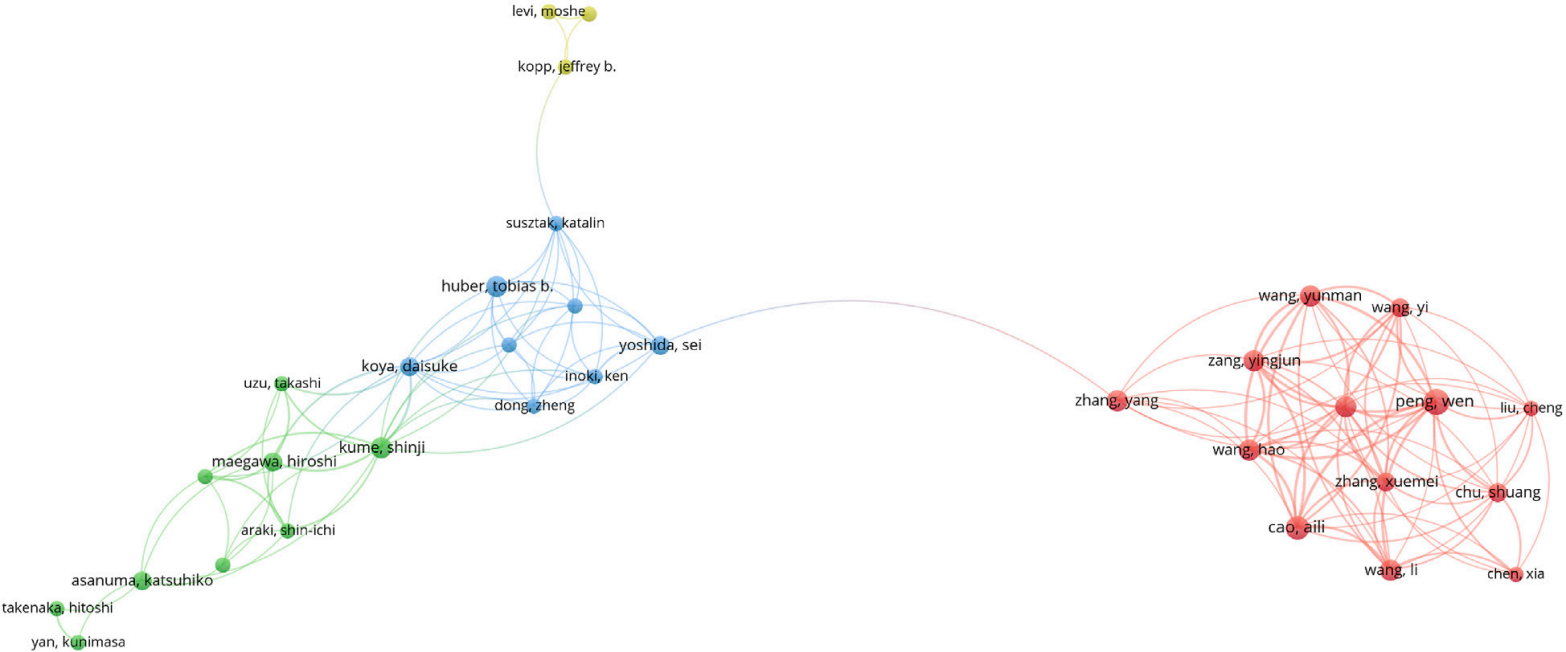
Author collaboration map.

In the field of glomerular podocytes and endoplasmic reticulum research, there are 1,679 authors involved in this field. The top 10 most productive authors are listed in Table 2. Total Link Strength (TLS) represents the number of co-occurrences of each country/institution or each author with other countries/institutions or authors, which to some extent reflects the collaborative communication relationship between countries/institutions or authors. And the collaboration between the authors is shown in Figures 5, 6, where a larger font size and greater yellow opacity signify a higher number of publications. The authors with the most publications are Cybulsky, Andrey V and Papillon, Joan, with 13 publications, followed by Guillemette, Julie (n = 8). The author collaboration graph reflects the collaborative relationships between authors, with authors in the same color scheme depicting their close collaboration, and the top 10 being cited more than 180 times, indicating that they are distinguished and influential researchers.

**TABLE 2:** Top 10 most prolific authors by publication count.

Rank	Author	Volume of publications	Citation frequency	Number of citations per article	TLS
1	Cybulsky, Andrey V	13	741	57.00	33
2	Papillon, Joan	13	197	15.15	47
3	Guillemette, Julie	8	112	14.00	34
4	Peng, Wen	6	271	45.17	39
5	Zhang, Chun	6	191	31.83	23
6	Takano, Tomoko	6	137	22.83	21
7	Sieber, Jonas	5	302	60.40	12
8	Mundel, Peter	5	290	58.00	12
9	Cao, Aili	5	189	37.80	35
10	Chen, Ying Maggie	5	180	36.00	16

#### 2.2.4 Journals and cited journals

The literature from the search was published in 140 journals, and the top 10 most prolific journals are listed in Table 3, the Journal of the American Society of Nephrology having the most articles (n = 17), followed by the American Journal of Physiology-renal Physiology (n = 14), followed by Kidney International (n = 12), indicating that these journals are highly contributing to the field. Meanwhile Journal of the American Society of Nephrology (n = 877) and Kidney International (n = 771), were the top 2 cited journals.

**TABLE 3:** Top 10 highly productive and co-cited journals.

Rank	Periodicals	Citations	If 2022	Rank	Co-cited journals	Citations	If 2022
1	Journal of the American Society of Nephrology	877	13.6	1	Journal of the American Society of Nephrology	1,221	13.6
2	American Journal of Physiology-renal Physiology	480	4.2	2	Kidney International	1,015	19.6
3	Kidney International	771	19.6	3	American Journal of Physiology-renal physiology	684	4.2
4	Scientific Reports	245	4.6	4	Journal of Biological Chemistry	619	4.8
5	Molecular Medicine Reports	99	3.4	5	Journal of Clinical Investigation	541	15.9
6	Frontiers in Pharmacology	70	5.6	6	Diabetes	457	7.7
7	Laboratory Investigation	215	5.0	7	Plos One	347	3.7
8	Journal of Biological Chemistry	154	4.8	8	Proceedings of the National Academy of Sciences of the United States of America	289	11.1
9	Plos One	336	3.7	9	American Journal of Pathology	288	6.0
10	Biochimica Et Biophysica Acta-molecular Basis of Disease	117	6.2	10	Nature	262	64.8

#### 2.2.5 Co-cited references and reference bursts

**FIGURE 7:**
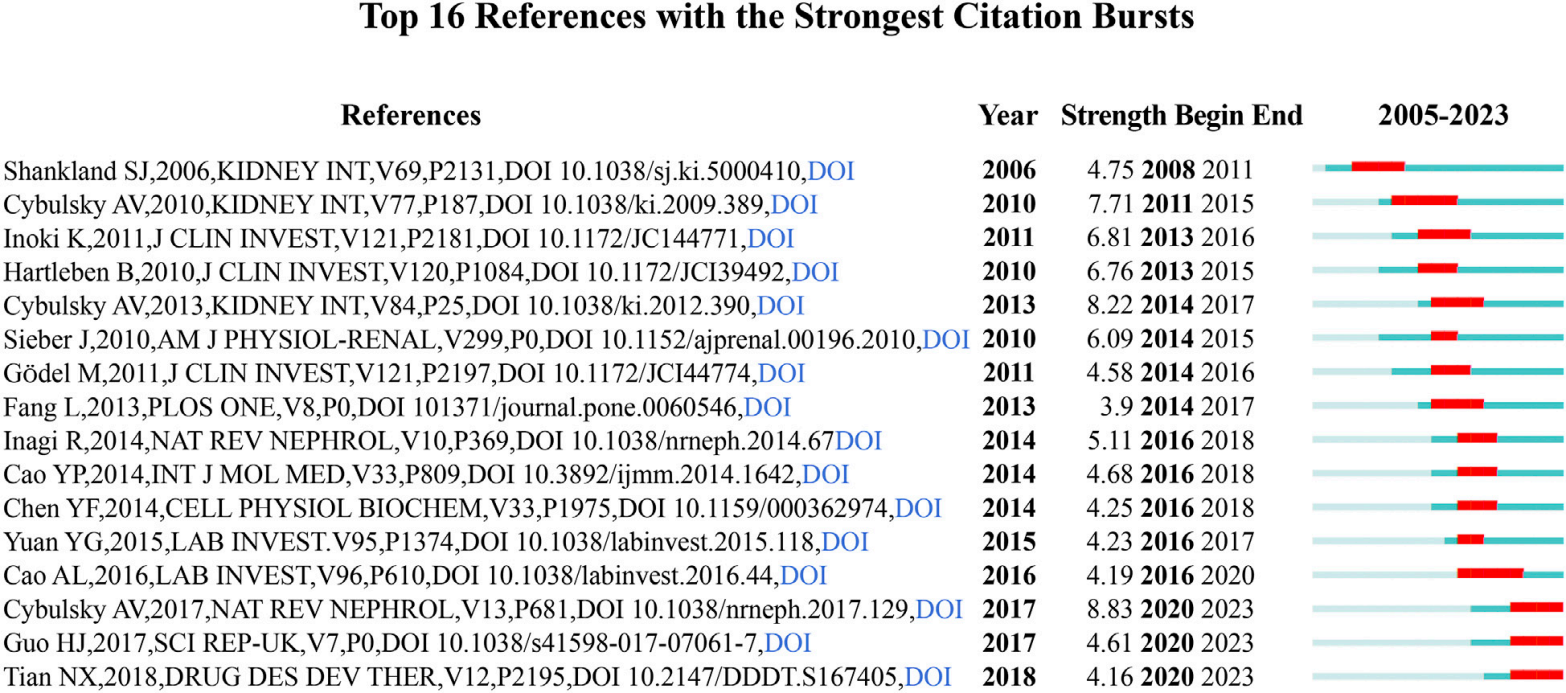
Top 16 references with the strongest citation bursts.

There were 13,361 co-citations in the literature resulting from the search, and Table 4 lists the 10 most co-cited articles, with the most co-cited article being Hartleben et al. (2010) published in 2010, which revealed that autophagy is an essential factor in maintaining glomerular podocyte homeostasis and determining glomerular podocyte senescence, and that autophagy may be a new target for the treatment of glomerular diseases. Followed by Lindenmeyer, Maja T (2008) published in 2008, which showed that insufficient endoplasmic reticulum stress leads to apoptosis, and that diabetes and proteinuria may lead to renal endoplasmic reticulum stress, but persistent hyperglycemia and proteinuria may lead to apoptosis. CiteSpace provides citation bursts of refrrences, meaning reference citations change significantly over time. In recent years, researchers have often cited references with citation bursts to represent emerging topics in specific research areas. Figure 7 shows the top 16 references with the strongest citation bursts. The most cited study is (Shankland, 2006) published in 2006 with a strength of 4.75, which discusses the causes of podocyte damage and loss, the link between changes in podocyte numbers and glomerulosclerosis, and suggests potential new therapies for the treatment of proteinuric nephropathy.

**TABLE 4:** Top 10 most frequently cited references with total citations.

Rank	Cited document	Citations	Total contact strength
1	Hartleben B., 2010, J Clin Invest, v120, p1084, doi 10.1172/jci39492	47	164.00
2	Lindenmeyer M. T., 2008, J Am Soc Nephrol, v19, p2225, doi 10.1681/asn.2007121313	40	178.00
3	Cybulsky A. V., 2010, Kidney Int, v77, p187, doi 10.1038/ki.2009.389	37	145.00
4	Cybulsky A. V., 2013, Kidney Int, v84, p25, doi 10.1038/ki.2012.390	37	130.00
5	Inoki K., 2011, J Clin Invest, v121, p2181, doi 10.1172/jci44771	37	157.00
6	Sieber J., 2010, Am J Physiol-renal, v299, pf821, doi 10.1152/ajprenal.00196.2010	36	137.00
7	Pavenstädt H., 2003, Physiol Rev, v83, p253, doi 10.1152/physrev.00020.2002	34	107.00
8	Mundel P., 1997, Exp Cell Res, v236, p248, doi 10.1006/excr.1997.3739	33	109.00
9	Cybulsky A. V., 2017, nat rev nephrol, v13, p681, doi 10.1038/nrneph.2017.129	31	77.00
10	Inagi R., 2005, Kidney Int, v68, p2639, doi 10.1111/j.1523-1755.2005.00736.x	28	128.00

#### 2.2.6 Research hot spots and frontier analysis

**FIGURE 8:**
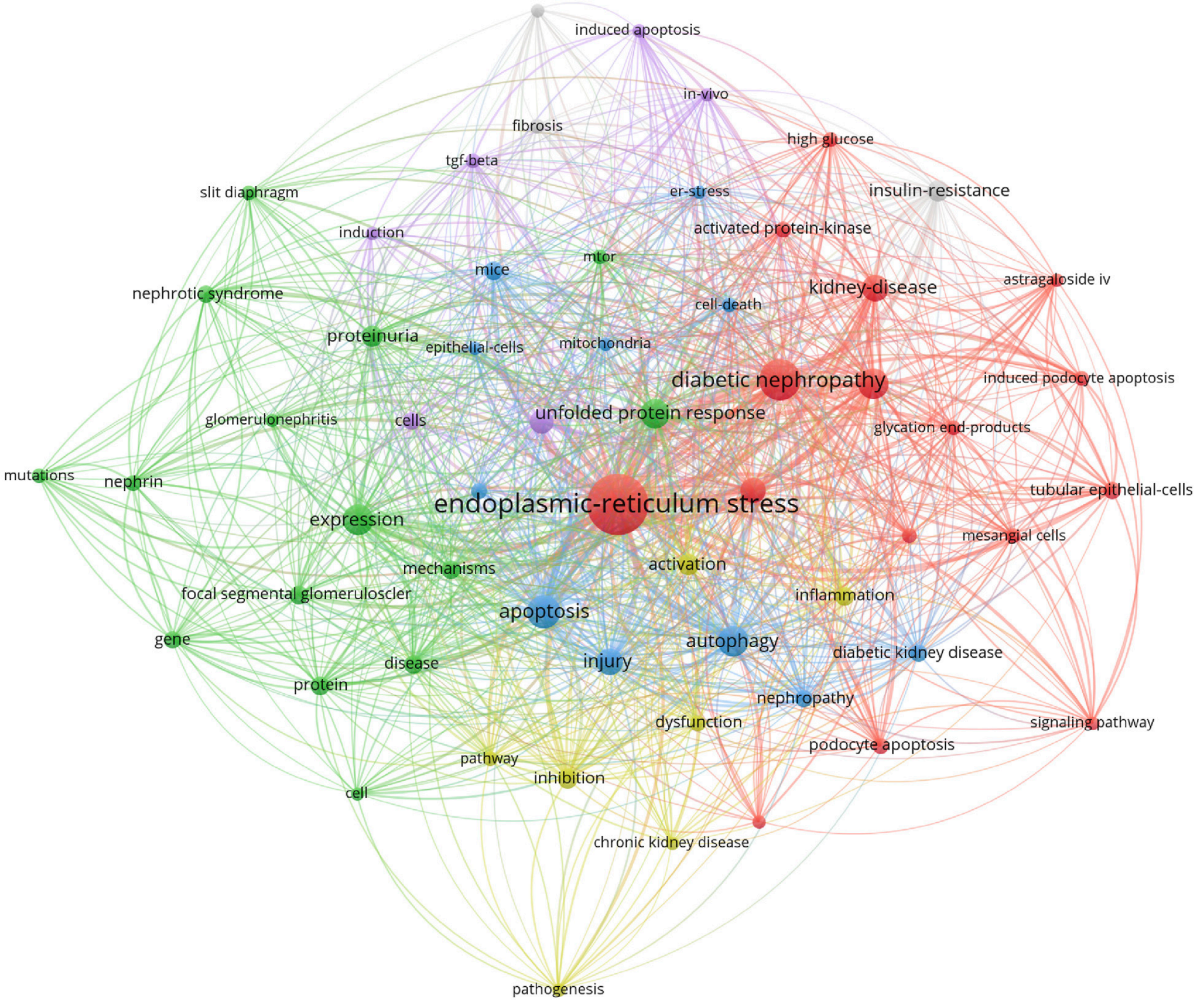
Keyword clustering graph.

**FIGURE 9:**
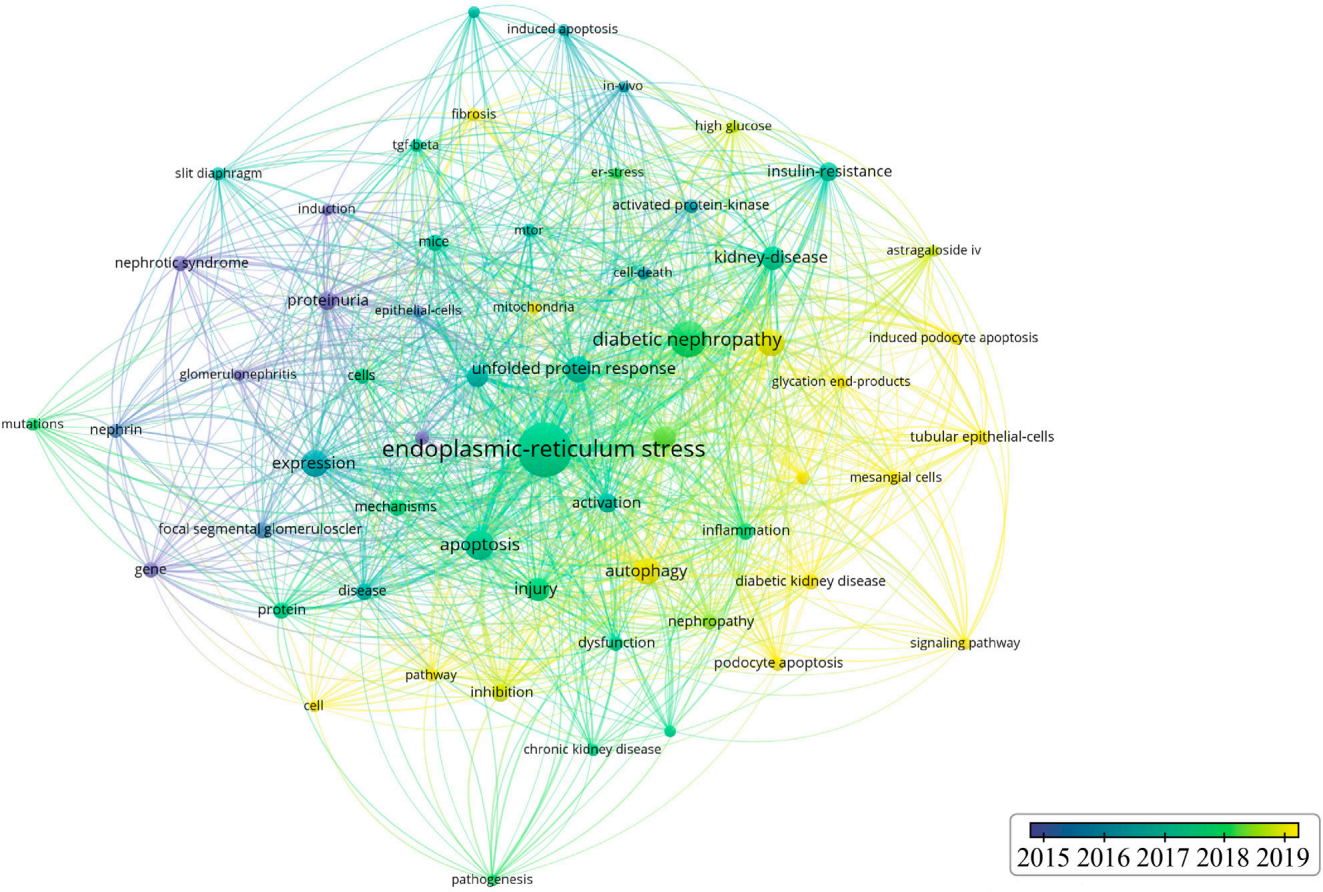
Keyword time graph.

**FIGURE 10:**
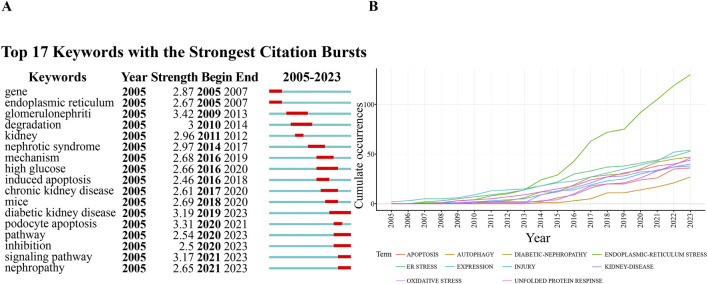
**(A)** Top 17 keywords with the highest citation bursts; **(B)** A visual dynamic map of the most commonly used keywords.

As shown in Table 5, the most frequently cited keywords in foot cell endoplasmic reticulum research over the past 19 years are endoplasmic reticulum stress (200), diabetic nephropathy (91), apoptosis (62), expression (54), autophagy (52), oxidative stress (50), unfolded protein response (49), injury (41), renal disease (38), and foot cell injury (36). These keywords reflect the research hotspots in the field. Out of 1,334 keywords, the minimum number of repetitions was set to 10 and 59 eligible keywords were selected for VOSviewer visualization and analysis. Each color in the network visualization graph represents a cluster, as in Figure 8, all the keywords can be classified into five clusters: the first cluster (red) is mainly about diabetic nephropathy, the second cluster (green) is nephropathy proteinuria, the third cluster (blue) is glomerular epithelial cell apoptosis, the fourth cluster (yellow) pathogenesis, and the fifth cluster (purple) TGF-BETA. Figure 9 indicates the trend of keywords over time. The yellow nodes indicate emerging themes, and in the last 5 years, advanced glycosylation end products, diabetic nephropathy, podocyte apoptosis, and cellular autophagy appeared with high frequency, suggesting that these aspects are the research hotspots in the field. In addition, we used CiteSpace for keyword citation burst detection. There are 17 keywords with strong bursts, as shown in Figure 10A. Gene ranked first and had the strongest burst (intensity = 2.87). The burst words in recent years were mainly diabetic nephropathy, podocyte apoptosis, cell signaling pathway, and inhibition, which are the current research frontiers of glomerular podocytes and endoplasmic reticulum. Figure 10B is a dynamic visualization of the authors’ most frequently used keywords. As shown, “endoplasmic-reticulum stress” has been growing rapidly and continuously, indicating that endoplasmic-reticulum stress has been a hotspot of endoplasmic reticulum research.

**TABLE 5:** Top 10 most frequently occurring keywords.

Rank	Keywords	Frequency	Total contact strength
1	endoplasmic-reticulum stress	200	912.00
2	diabetic nephropathy	91	475.00
3	apoptosis	62	340.00
4	expression	54	247.00
5	autophagy	52	274.00
6	oxidative stress	50	268.00
7	unfolded protein response	49	242.00
8	injury	41	203.00
9	kidney-disease	38	195.00
10	podocyte injury	36	170.00

## 3 Discussion

This study reveals relevant findings and advances in the glomerular podocyte endoplasmic reticulum research. Primarily, 275 articles from 1 January 2005 to 22 October 2023 were analyzed. Currently, 437 institutions from 46 countries are actively involved in the field, with China being the main contributor of academic articles, followed by the United States, Japan and Canada. It is noteworthy that the United States exhibits the greatest intensity of total linkages. It maintains close partnerships with many countries, including China, Japan, and Canada, where cross-border institutional collaboration can contribute to scientific progress. In terms of publication and research quality, China boasts the highest level of research. However, the United States is the most frequently cited country, with China a close second. McGill University was the most published and most cited institution. In terms of article creation, Cybulsky, Andrey V has the highest number of publications at 13, while Sieber, Jonas is the author with the highest average number of citations.

### 3.1 Research status

We analyzed and integrated keywords using various software tools. Through this process, we identified several emerging research trends in the field. These trends include “endoplasmic reticulum stress,” “unfolded protein response,” “diabetic nephropathy,” “autophagy,” “apoptosis” and “cell signaling pathways.” These aspects are summarized in the following sections.

#### 3.1.1 Endoplasmic reticulum stress and the unfolded protein response

The endoplasmic reticulum (ER) is an essential site for protein folding and maturation in eukaryotic cells. Podocytes are highly sensitive to ER stress. Hyperglycemia, persistent proteinuria and free fatty acids can amplify or induce ER stress-induced apoptosis, resulting in podocyte injury (Fang et al., 2013; Lindenmeyer et al., 2008; Sieber et al., 2010). The cellular requirements for synthesizing proteins within the endoplasmic reticulum are balanced with its folding capacity. However, physiological demands or aberrations in folding can lead to an imbalance, resulting in the accumulation of misfolded proteins. Accumulation of misfolded proteins in the ER leads to endoplasmic reticulum stress, which activates the unfolded protein response (UPR) to ensure correct protein synthesis. The UPR is activated to ensure correct protein synthesis (Marciniak et al., 2022). The UPR is an endogenous stress response signaling pathway pathway that consisting of three ER-localized transmembrane The UPR consists of three integrated signaling pathways emanating from ER-localized transmembrane proteins:inositol-requiring enzyme 1α (IRE1α), PKR-like endoplasmic reticulum kinase (PERK), and activating transcription factor 6 (ATF6). Heavy-chain binding protein (BiP) is a critical endoplasmic reticulum regulatory protein that normally keeps all three protein genes inactive in quiescent cells. The induction of endoplasmic reticulum stress may be protective or injurious. When there are too many misfolded proteins in the ER, the UPR can reset the folding capacity of the ER to restore the ER protein homeostasis. However, when endoplasmic reticulum stress persists at high levels for an extended period of time, the UPR sends pro-inflammatory and pro-death signals that lead to cell death. Research is ongoing into the factors that control the transition from an adaptive to a pro-apoptotic unfolded protein response, yet the factors are poorly understood (Navarro-Betancourt et al., 2022). Protein misfolding and endoplasmic reticulum stress are evident in the renal diseases of primary glomerulonephritis, glomerulopathy associated with genetic mutations, diabetic nephropathy, acute kidney injury, chronic kidney disease, and renal fibrosis. Chronic endoplasmic reticulum stress-induced cellular damage is becoming a critical factor in human diseases, including diabetes, neurodegenerative diseases and cancer (Hetz, 2012). Activation of the endoplasmic reticulum stress response pathway has been observed in diabetes and various cancers (Schwarz and Blower, 2015). How the endoplasmic reticulum stress response pathway functions in these pathologies is an active area of research, and various components of the stress response pathway are being investigated as potential therapeutic targets (Ryno et al., 2013). Additionally, the issue of how to preserve the equilibrium of ER stress is a matter that warrants consideration.

#### 3.1.2 Podocyte autophagy and endoplasmic reticulum stress

Autophagy, first proposed by Christian de Duve in 1963, is a highly conserved intracellular protein degradation process (Ravikumar et al., 2010). It transports proteins and other damaged organelles to the lysosome for degradation and recycling, thereby maintaining continuous organelle renewal and intracellular homeostasis (Mizushima et al., 2010). Autophagy can be divided into macro-autophagy, micro-autophagy and partner-mediated autophagy. At the same time, macroautophagy can be divided into endoplasmic reticulum autophagy, mitophagy, lipid autophagy and so on (Yang M. et al., 2023). The decrease in autophagy may exacerbate the level of damage in podocyte disorders, including focal segmental glomerulosclerosis, and could potentially worsen podocyte injury in elderly individuals affected by podocyte diseases. The key factor in regulating autophagy is the autophagy-related gene 5 (Atg5), and its specific deletion can manifest as a reduction in autophagy and a decrease in podocytes (Zhao et al., 2024). However, sustained autophagy in renal tubular cells promotes renal fibrosis, indicating that autophagy is a double-edged sword (Lin et al., 2021). Autophagosomes are mainly located on the epithelial side of the glomerular basement membrane where the podocytes are located. The endoplasmic reticulum is the main source of membranes supplying autophagosome biogenesis, and ER-phagocytosis is considered as a new method to regulate endoplasmic reticulum homeostasis (Yang M. et al., 2023). Hartleben et al. (2010) reported that autophagy affects glomerular disease susceptibility and maintains podocyte homeostasis in aged mice. Kouroku et al. (2006) proposed that autophagy formation is a cellular defense mechanism that counteracts polyQ72-induced ER stress-mediated cell death through degradation of amplified polyglutamine 72 repeat sequence (polyQ72) aggregates. Currently, there are limited studies on endoplasmic reticulum stress and autophagy markers in podocyte and podocyte diseases, and the signaling pathways linking endoplasmic reticulum stress to autophagy, the mechanism of selecting the endoplasmic reticulum as an autophagic cargo, the crosstalk between the endoplasmic reticulum stress-induced autophagy and cell death pathways, and the impact of autophagy in endoplasmic reticulum stress-associated diseases remain largely unanswered.

#### 3.1.3 Association of podocytes with diabetic nephropathy

Diabetes affects all cell types in the kidney, including podocytes. Diabetic nephropathy (DN) is a glomerular proteinuria disease of diabetes mellitus and is the leading cause of end-stage renal disease (ESRD). ER stress and oxidative stress induced by hyperglycemia may be the cause of DKD (Yang C. et al., 2023). On the one hand, hyperglycemia inhibits the cytoprotective effects of ER stress in foot cells and amplifies the pro-apoptotic effects of endoplasmic reticulum stress in foot cells, resulting in foot cell damage (Fang et al., 2013). Cybulsky et al. (2024) proposed that mice with podocyte-specific deletion of IRE1α demonstrate more severe diabetic nephropathy. On the other hand, hyperglycemia induces the production of reactive oxygen species (ROS), which leads to the disappearance of podocytes and detachment from GBM or death, further increasing the permeability of the glomerular filtration barrier to plasma proteins, thereby increasing proteinuria (Lin and Susztak, 2016; Barutta et al., 2022). Oleanolic acid (OA) and N-acetylcysteine (NAC) have therapeutic effects in DN through reduction of oxidative stress and ER stress (Lee et al., 2015). Therefore, the development of DN is closely related to the loss and death of podocytes. The pattern of death of podocytes in DN includes apoptosis, autophagy, mitotic catastrophe (MC), anaplasia, necrotic apoptosis and pyroptosis (Jiang et al., 2022). In DN, massive apoptosis of podocytes leads to excessive loss of podocytes, making it difficult to maintain glomerular structure and function. Most of the urinary podocytes in diabetic patients show MC rather than apoptosis. Therefore, MC is the main cause of podocyte loss (Hara et al., 2019). Activation of mammalian target of rapamycin complex 1 (mTORC1) induces the UPR to disrupt the regulatory system of energy metabolism. MTORC1 hyperactivation stimulates ERS- and EMT-like phenotypes in podocytes, which ultimately leads to podocyte weakness, and decreasing podocyte mTORC1 activity is a potential therapeutic strategy to prevent DN (Inoki et al., 2011). Yasuda-Yamahara et al. (2015) suggested that inadequate podocyte autophagy is associated with the progression of DN and proposed that autophagy activation may be a therapeutic target in diabetic patients with significant proteinuria and rapidly declining renal function. Madhusudhan et al. (2015) demonstrated that IRE1/XBP1-dependent UPR is essential for maintaining podocyte homeostasis and function. They also showed that impaired insulin signaling hampers XBP1 activity in podocytes and exacerbates ATF6-dependent maladaptive ER responses in diabetic mice. Endoplasmic reticulum stress inhibitor TUDCA not only reduces proteinuria in diabetic mice but also alleviates podocyte damage (Yang C. et al., 2023). Therefore, reducing ER stress is a potential therapeutic strategy for DN targeting ER pathways. ER stress accelerates the progression of DN by damaging podocytes, mesangial cells and endothelial cells (Wang et al., 2022). However, in the diabetic state, the factors that stimulate endoplasmic reticulum stress and their precise roles in podocytes remain unclear. The mechanism by which endoplasmic reticulum stress is activated in DN also unclear. These require further investigation.

## 4 Conclusion

This paper analyzes the hotspots, frontiers and trends in research on glomerular podocytes and endoplasmic reticulum through trends in keyword clustering, bursts and timeline relationships based on a bibliometric analysis constructed with Citespace, VOSviewer, the online platform for metrics literature and the Bibliometrix software package. The research literature on the has increased significantly in recent decades, indicating a growing interest in this emerging field. Our findings comprehensively summarize the current status of endoplasmic reticulum research in glomerular podocytes and are of great significance for future research efforts.

## Data Availability

The original contributions presented in the study are included in the article/supplementary material, further inquiries can be directed to the corresponding author.

## References

[B1] Almanza A. Carlesso A. Chintha C. Creedican S. Doultsinos D. Leuzzi B. (2018). Endoplasmic reticulum stress signalling - from basic mechanisms to clinical applications. FEBS J. 286 (2), 241–278. 10.1111/febs.14608 30027602 PMC7379631

[B2] Barutta F. Bellini S. Gruden G. (2022). Mechanisms of podocyte injury and implications for diabetic nephropathy. Clin. Sci. 136 (7), 493–520. 10.1042/cs20210625 PMC9008595 35415751

[B3] Cybulsky A. V. Papillon J. Guillemette J. Navarro-Betancourt J. R. Chung C.-F. Iwawaki (2024). Deletion of IRE1α in podocytes exacerbates diabetic nephropathy in mice. Sci. Rep. 14 (1), 11718. 10.1038/s41598-024-62599-7 38778209 PMC11111796

[B4] Fang L. Zhou Y. Cao H. Wen P. Jiang L. He W. (2013). Autophagy attenuates diabetic glomerular damage through protection of hyperglycemia-induced podocyte injury. PLOS ONE 8 (4), e60546. 10.1371/journal.pone.0060546 23593240 PMC3623813

[B5] Hara M. Oohara K. Dai D.-F. Liapis H. (2019). Mitotic catastrophe causes podocyte loss in the urine of human diabetics. Am. J. Pathology 189 (2), 248–257. 10.1016/j.ajpath.2018.10.016 PMC6943371 30472210

[B6] Hartleben B. Gödel M. Meyer-Schwesinger C. Liu S. Ulrich T. Sven K. (2010). Autophagy influences glomerular disease susceptibility and maintains podocyte homeostasis in aging mice. J. Clin. Invest. 120 (4), 1084–1096. 10.1172/jci39492 20200449 PMC2846040

[B7] Hetz C. (2012). The unfolded protein response: controlling cell fate decisions under ER stress and beyond. Nat. Rev. Mol. Cell Biol. 13 (2), 89–102. 10.1038/nrm3270 22251901

[B8] Inoki K. Mori H. Wang J. Suzuki T. Hong S. Yoshida S. (2011). mTORC1 activation in podocytes is a critical step in the development of diabetic nephropathy in mice. J. Clin. Invest. 121 (6), 2181–2196. 10.1172/jci44771 21606597 PMC3104745

[B9] Jiang A. Song A. Zhang C. (2022). Modes of podocyte death in diabetic kidney disease: an update. J. Nephrol. 35 (6), 1571–1584. 10.1007/s40620-022-01269-1 35201595

[B10] Kopp J. B. Anders H. J. Susztak K. Podestà M. A. Remuzzi G. Hildebrandt F. (2020). Podocytopathies. Nat. Rev. Dis. Prim. 6 (1), 68. 10.1038/s41572-020-0196-7 32792490 PMC8162925

[B11] Kouroku Y. Fujita E. Tanida I. Ueno T. Isoai A. Kumagai H. (2006). ER stress (PERK/eIF2alpha phosphorylation) mediates the polyglutamine-induced LC3 conversion, an essential step for autophagy formation. Cell Death & Differ. 14 (2), 230–239. 10.1038/sj.cdd.4401984 16794605

[B12] Lee E. S. Kim H. M. Kang J. S. Lee E. Y. Yadav D. Kwon M.-H. (2015). Oleanolic acid andN-acetylcysteine ameliorate diabetic nephropathy through reduction of oxidative stress and endoplasmic reticulum stress in a type 2 diabetic rat model. Nephrol. Dial. Transplant. 31 (3), 391–400. 10.1093/ndt/gfv377 26567248

[B13] Lin J. S. Susztak K. (2016). Podocytes: the weakest link in diabetic kidney disease? Curr. diabetes Rep. 16 (5), 45. 10.1007/s11892-016-0735-5 PMC5064850 27053072

[B14] Lin Q. Banu K. Ni Z. Leventhal J. S. Menon M. C. (2021). Podocyte autophagy in homeostasis and disease. J. Clin. Med. 10 (6), 1184. 10.3390/jcm10061184 33809036 PMC7998595

[B15] Lindenmeyer M. T. Rastaldi M. P. Ikehata M. Neusser M. A. Kretzler M. Cohen C. D. (2008). Proteinuria and hyperglycemia induce endoplasmic reticulum stress. J. Am. Soc. Nephrol. 19 (11), 2225–2236. 10.1681/asn.2007121313 18776125 PMC2573014

[B16] Madhusudhan T. Wang H. Dong W. Ghosh S. Bock F. Thangapandi V. R. (2015). Defective podocyte insulin signalling through p85-XBP1 promotes ATF6-dependent maladaptive ER-stress response in diabetic nephropathy. Nat. Commun. 6 (1), 6496. 10.1038/ncomms7496 25754093 PMC4366504

[B17] Marciniak S. J. Chambers J. E. Ron D. (2022). Pharmacological targeting of endoplasmic reticulum stress in disease. Nat. Rev. Drug Discov. 21 (2), 115–140. 10.1038/s41573-021-00320-3 34702991

[B18] Mizushima N. Yoshimori T. Levine B. (2010). Methods in mammalian autophagy research. Cell 140 (3), 313–326. 10.1016/j.cell.2010.01.028 20144757 PMC2852113

[B19] Navarro-Betancourt J. R. Papillon J. Guillemette J. Iwawaki T. Chung C.-F. Cybulsky A. V. (2022). The IRE1α pathway in glomerular diseases: the unfolded protein response and beyond. Front. Mol. Med. 9, 824417. 10.3389/fmolb.2022.824417 PMC11285563 39086958

[B20] Oakes S. A. Papa F. R. (2015). The role of endoplasmic reticulum stress in human pathology. Annu. Rev. pathology 10, 173–194. 10.1146/annurev-pathol-012513-104649 PMC5568783 25387057

[B21] Pavenstädt H. Kriz W. Kretzler M. (2003). Cell biology of the glomerular podocyte. Physiol. Rev. 83 (1), 253–307. 10.1152/physrev.00020.2002 12506131

[B22] Ravikumar B. Sarkar S. Davies J. E. Futter M. Garcia-Arencibia M. Green-Thompson Z. W. (2010). Regulation of mammalian autophagy in Physiology and pathophysiology. Physiol. Rev. 90 (4), 1383–1435. 10.1152/physrev.00030.2009 20959619

[B23] Roumeliotis S. Liakopoulos V. Veljkovic A. Dounousi E. (2023). Redox systems biology in chronic kidney disease. Oxidative Med. Cell. Longev. 2023, 1–3. 10.1155/2023/9864037 PMC10171981 37180759

[B24] Ryno L. M. Wiseman R. L. Kelly J. W. (2013). Targeting unfolded protein response signaling pathways to ameliorate protein misfolding diseases. Curr. Opin. Chem. Biol. 17 (3), 346–352. 10.1016/j.cbpa.2013.04.009 23647985 PMC5859939

[B25] Schwarz D. S. Blower M. D. (2015). The endoplasmic reticulum: structure, function and response to cellular signaling. Cell. Mol. Life Sci. 73 (1), 79–94. 10.1007/s00018-015-2052-6 26433683 PMC4700099

[B26] Shankland S. J. (2006). The podocyte’s response to injury: role in proteinuria and glomerulosclerosis. Kidney Int. 69 (12), 2131–2147. 10.1038/sj.ki.5000410 16688120

[B27] Sieber J. Lindenmeyer M. T. Kampe K. Campbell K. A. Cohen C. D. Hopfer H. (2010). Regulation of podocyte survival and endoplasmic reticulum stress by fatty acids. Am. J. Physiol. Ren. Physiol. 299 (4), F821–F829. 10.1152/ajprenal.00196.2010 PMC2957252 20668104

[B28] Wang X. Zhao J. Li Y. Rao J. Xu G. (2022). Epigenetics and endoplasmic reticulum in podocytopathy during diabetic nephropathy progression. Front. Immunol. 13, 1090989. 10.3389/fimmu.2022.1090989 36618403 PMC9813850

[B29] Yang C. Zhang Z. Liu J. Chen P. Li J. Shu H. (2023a). Research progress on multiple cell death pathways of podocytes in diabetic kidney disease. Mol. Med. 29 (1), 135. 10.1186/s10020-023-00732-4 37828444 PMC10571269

[B30] Yang M. Liu C. Jiang N. Liu Y. Luo S. Li C. (2023b). Endoplasmic reticulum homeostasis: a potential target for diabetic nephropathy. Front. Endocrinol. 14, 1182848. 10.3389/fendo.2023.1182848 PMC10296190 37383398

[B31] Yasuda-Yamahara M. Kume S. Tagawa A. Maegawa H. Uzu T. (2015). Emerging role of podocyte autophagy in the progression of diabetic nephropathy. Autophagy 11 (12), 2385–2386. 10.1080/15548627.2015.1115173 26565953 PMC4835189

[B32] Zhao Q. Huang Y. Fu N. Cui C. Peng X. Kang H. (2024). Podocyte senescence: from molecular mechanisms to therapeutics. Ren. Fail. 46 (2), 2398712. 10.1080/0886022x.2024.2398712 39248407 PMC11385655

